# Molecular Epidemiology of *Theileria annulata* in Cattle from Two Districts in Punjab (Pakistan)

**DOI:** 10.3390/ani11123443

**Published:** 2021-12-02

**Authors:** Asia Parveen, Abeer Mousa Alkhaibari, Muhammad Asif, Hamdan I. Almohammed, Zahra Naqvi, Adil Khan, Munir Aktas, Sezayi Ozubek, Muhammad Farooq, Furhan Iqbal

**Affiliations:** 1 Institute of Pure and Applied Biology, Zoology Division, Bahauddin Zakariya University, Multan 60800, Pakistan; asiaiqbal790@gmail.com (A.P.); ranaasifs786@gmail.com (M.A.); zahranaqvi154@gmail.com (Z.N.); 2 Department of Biology, Faculty of Science, University of Tabuk, Tabuk 71491, Saudi Arabia; aalkhaibari@ut.edu.sa; 3 Department of Microbiology and Parasitology, Almaarefa University, Riyadh 11597, Saudi Arabia; hamohammed@mcst.edu.sa; 4 Department of Zoology, Abdul Wali Khan University, Mardan 23200, Pakistan; zoologyawkum@gmail.com; 5 Department of Parasitology, Veterinary Faculty, University of Firat, Elazig 23119, Turkey; maktas@firat.edu.tr (M.A.); sozubek@firat.edu.tr (S.O.); 6 Department of Zoology, Ghazi University, Dera Ghazi Khan 32200, Pakistan

**Keywords:** *Theileria annulata*, *cytochrome b* gene, *30 kDa* gene, epidemiology, phylogenetic analysis

## Abstract

**Simple Summary:**

Pakistan is a tropical country where climate is favourable for tick growth and hence its incidence of tick-borne diseases is high, affecting the output of the livestock sector. In the present study the infection rate of *Theileria annulata*, the causative agent of bovine theileriosis, was compared in apparently healthy cattle enrolled from two different regions in Pakistan. Parasite prevalence was found to be higher in Dera Ghazi Khan District than in Lodhran. The infection rate was higher in cattle that were infested with ticks and in those animals that were housed indoors at dairy farms with other animals. This prophylactic detection of parasite will help to design strategies to control tick and tick-borne diseases in study areas.

**Abstract:**

The present study was designed to report the molecular prevalence of *T. annulata* in cattle blood samples collected from Punjab in Pakistan. A total of 428 cattle blood samples were collected from Districts Lodhran (*n* = 218) and Dera Ghazi Khan (*n* = 210). The prevalence of *T. annulata* was determined by the amplification of a fragment from its *cytochrome b* gene and parasite prevalence was significantly higher (*p* = 0.03) in the blood samples of cattle collected from Dera Ghazi Khan (70/210; 33%) as compared to Lodhran (52/218; 24%). Presence of *T. annulata* was also confirmed by the amplification of a fragment from their *30 kDa* gene. The amplified PCR products of both genes were confirmed by DNA sequencing and these partial DNA sequences were submitted to GenBank. Phylogenetic analysis revealed that amplified partial gene sequences resembled previously reported *T. annulata* sequences in cattle from India, China, Iran, Tunisia, Turkey and Egypt. The incidence of *T. annulata* infection was higher in Sahiwal cattle (*p* = 0.04) than the other enrolled cattle breed from Dera Ghazi Khan. Female cattle from Lodhran (*p* = 0.02), while males (*p* = 0.02), animals housed in close compounds (*p* = 0.04), animals with a tick burden (*p* = 0.005) and farms with only cattle (*p* = 0.01) in Dear Ghazi Khan were found to be more susceptible to *T. annulata* infection. We recommend that large-scale tick and tick-borne disease control strategies be implemented in both districts under investigation, especially in Dera Ghazi Khan.

## 1. Introduction

The livestock and dairy industry faces many challenges in Pakistan, including a lack of awareness among livestock owners regarding feed, tick management, artificial insemination, financial constraints and the limited availability of health facilities [1]. Parasitism is a crucial problem causing health problems in livestock farms. Ticks are one of the most important ectoparasites in this region. This is favoured by the climate in Pakistan, which provides optimal conditions for the development and reproduction of ticks [2]. A large variety of ticks (belonging to genera *Hyalomma, Rhipicephalus* and *Ixodes*) have been reported in Pakistan that infest a variety of domestic and wild animals, causing a variety of tick-borne diseases [3]. Theileriosis is one of the more common diseases affecting bovine health and productivity, causing economic losses to livestock owners [2]. *Theileria annulata,* an intracellular protozoan parasite, is the causative agent of bovine theileriosis [4]. *Theileria* spp. are known to be transmitted by a variety of Ixodid ticks that belong to genera *Rhipicephalus*, *Hyalomma*, *Amblyomma* and *Haemaphysalis* [5]. During the life cycle, the sporogony and merogony stages occur in bovine hosts while zygotes and kinetes are formed in ticks [6]. During tick feeding, parasites enter the host and rapidly invade its leukocytes. Ultimately merozoites are produced and released from the infected leukocytes, then entering into erythrocytes and developing into piroplasms [7]. Theileriosis is characterized by high fever, weakness, weight loss, inappropriate appetite, conjunctival petechiae, enlarged lymph nodes and anaemia [8]. Theileriosis is treated by oral implementation of halofuginone and by intra-muscular infusion of buparvaquone [9]. Animals surviving from acute disease usually become carriers of *Theileria* piroplasms and act as reservoirs for the maintenance of the parasite population [10]. Therefore, identification of carrier animals is worthwhile in epidemiological studies for concluding the infection risk, and for the implementation and monitoring of control programs [11]. The present study was designed for the molecular epidemiology and phylogenetic analysis of *T. annulata* in blood samples of various cattle breeds collected from two regions of South Punjab.

## 2. Materials and Methods

### 2.1. Study Areas and Subjects

**Figure 1:**
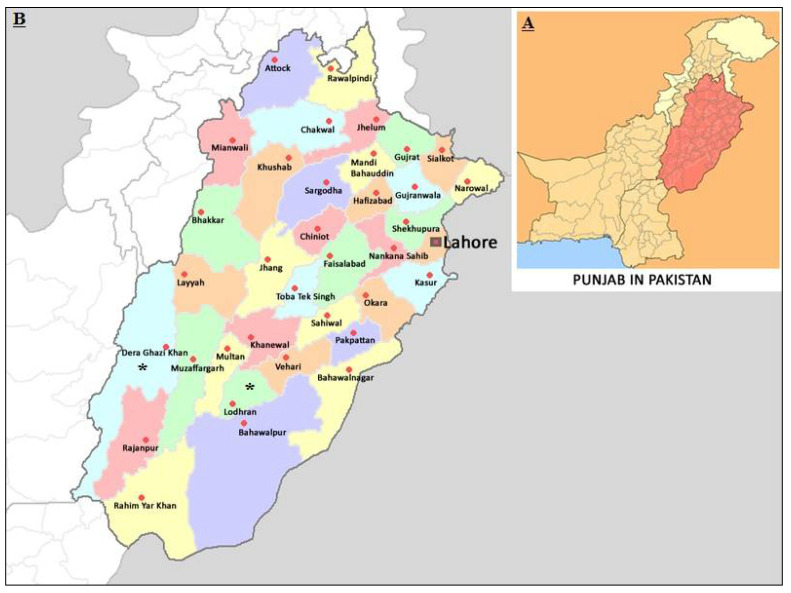
(**A**) Map of Pakistan with Punjab province highlighted. (**B**) Districts Lodhran and Dera Ghazi Khan, from where cattle blood samples were collected, are marked with * in the magnified section.

Two geographically different but important livestock-rich regions (Dera Ghazi Khan and Lodhran) from South Punjab, Pakistan, were selected for blood sampling and to compare the prevalence of *T. annulata* between them during current investigation (Figure 1). Lodhran District is located at 29°32′34″ N and 71°37′48″ E and Dera Ghazi Khan is located at 30°1′59″ N and 70°38′24″ E. The overall climate of both regions is hot and dry with little rainfall. The winter is mild with an average temperature of 4 °C, but summer is very hot with an average temperature of 42 °C.

### 2.2. Sample Collection and DNA Extraction

A total of 428 blood samples were collected from apparently healthy cattle from Districts Lodhran (*n* = 218) and Dera Ghazi Khan (*n* = 210) from June to October 2019 (the tick season). Approximately 3–5 mL of blood was collected from the jugular vein of each animal in a EDTA containing tube following informed consent from their owners. Blood was later used to extract DNA by using a QIAamp DNA kit (Qiagen, Germany) following the manufacturer’s instructions. A predesigned questionnaire was used to collect data regarding breed, sex, animal age, disease history, presence of dogs in the herds, tick burdens on cattle and dogs, placement of animals in farms and water supply to farms.

### 2.3. PCR Amplification of Cytochrome b and 30 kDa Gene

For the PCR amplification of a fragment from the mitochondrial *cytochrome b* gene of *T. annulata,* a set of primers were used (F 5′ ACTTTGGCCGTAATGTTAAAC 3′ and R 5′ CTCTGGACCAACTGTTTGG 3′), as described by Bilgic et al. [12]. The reaction mixture was prepared in a final volume of 25 µL consisting of 13 mM Tris–HCl (pH 8.3), 65 mM KCl, 2 mM MgCl_2_, 300 µM of each dNTP, 1 U of AmpliTaq DNA polymerase, 0.5 µM of each primer and 2 µL of template DNA and distilled sterile water to 25 µL volume. Reaction conditions comprised an initial denaturation step at 94 °C for 5 min followed by 30 cycles of denaturation at 95 °C for 50 s, primer annealing at 56 °C for 50 s and extension at 65 °C for 50 s. A final extension at 65 °C for 5 min was performed [12].

A fragment from the *30 kDa* merozoite surface antigen of *T. annulata* was amplified using oligonucleotide primers (N516: F 5′ GTAACCTTTAAAAACGT 3′ and N517: R 5′ GTTACGAACATGGGTTT 3′) as described by d’Oliveira et al. [13]. The final volume of the reaction mixture was 25 µL, which contained 50 mM KCl, 10 mM Tris-HCl (pH 8.3), 1.5 mM MgCl_2_, 0.1% Triton X-100, 200 µM (each) deoxynucleotide triphosphate, 2.5 U of Taq DNA polymerase (Merck, Kenilworth, NJ, USA), 20 pMol of primers and 5 µL of extracted DNA sample [13]. The thermocycler profile consisted of 94 °C for 5 min, 35 cycles of 94 °C for 30 s, 55 °C for 40 s, 72 °C for 45 s and final extension at 72 °C for 10 min [14]. *T. annulata* positive sample (from our previous study) and negative samples (reaction mixture without DNA but distilled sterile water to get the same volume, and so the same reaction conditions) were amplified during each PCR reaction as positive and negative controls, respectively.

### 2.4. DNA Sequencing and Phylogenetic Analysis of Cytochrome b and 30 kDa Gene

To confirm the PCR results, 7 amplified fragments (4 of *cytochrome b* and 3 of *30 kDa* gene) of *T. annulata* were randomly selected and DNA sequenced by a commercial lab (First Base Sequencing Service, Selangor, Malaysia) with the same primers as used for the PCR. The obtained DNA sequences were assessed on nucleotide BLAST to check their similarity index and they were registered in GenBank.

**Figure 2:**
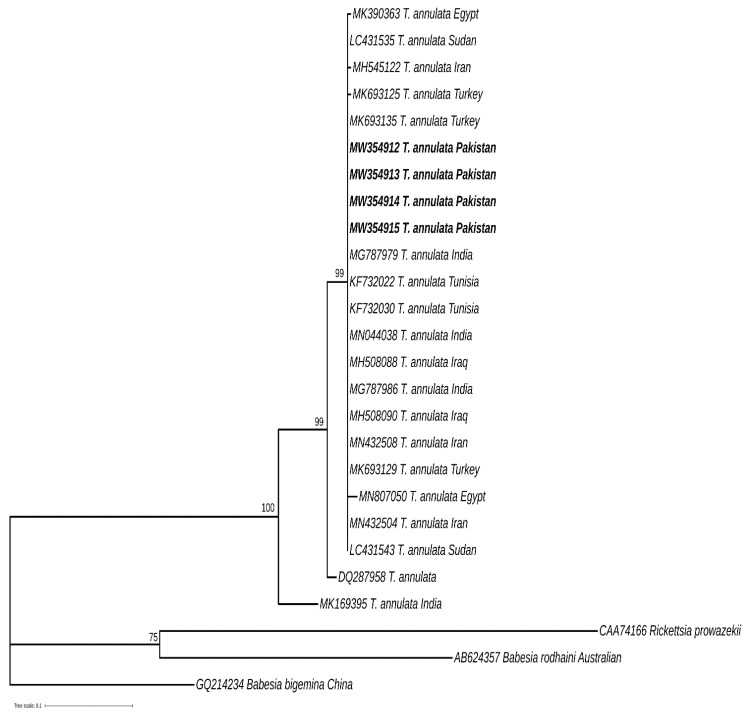
Phylogenetic tree based on partial *cytochrome b* gene sequences from *T. annulata* isolates from cattle in Pakistan and cattle worldwide, available in GenBank. The evolutionary history was inferred using the maximum likelihood method with the Hasegawa–Kishino–Yano model. Four new sequences of *T. annulata* obtained in the present study are represented in bold. The scale bar represents 0.2 substitutions per nucleotide position.

For the *cytochrome b* gene, 22 related sequences were obtained from GenBank and trimmed to 284 bp for sequence comparison. The phylogenetic reconstruction approach was managed in MEGA version X (MEGA, Philadelphia, PA, USA) [15] by using the maximum likelihood method with the Hasegawa–Kishino–Yano model [16]. Bootstrapping of 1000 replicates was conducted for confidence support. The phylogenetic tree was edited online with iTOL software (iTOL, Liverpool, UK). Sequences elucidated in this work are shown in bold font and GenBank access numbers are indicated for all entries. Values at the nodes represent the number of occurrence of clades in 1000 bootstrap replicates and branches with values less than 70% were eliminated (Figure 2).

For the *30 kDa* gene, the evolutionary history was inferred using the maximum likelihood method and the Tamura 3-parameter model [17]. The percentage of trees in which the associated taxa clustered together is shown next to the branches. Initial tree(s) for the heuristic search were obtained automatically by applying neighbour-joining and BioNJ algorithms to a matrix of pairwise distances estimated using the Tamura 3-parameter model and then selecting the topology with superior log likelihood value. A discrete gamma distribution was used to model evolutionary rate differences among sites (5 categories (+G, parameter = 0.1471)). The tree was drawn to scale, with branch lengths measured in the number of substitutions per site. This analysis involved 28 nucleotide sequences. Codon positions included were 1st + 2nd + 3rd + Noncoding. There was a total of 858 positions in the final dataset. Evolutionary analyses were conducted in MEGA X [15].

### 2.5. Statistical Analysis

The statistical package Minitab (version 16, State College, Pennsylvania, USA) was used for the statistical analysis. Animals were grouped into two age categories: animals up to five years of age (young) and more than five years (mature). The association between the presence of *T. annulata* and studied epidemiological factors was assessed by contingency table analysis using the Fisher’s exact test (for 2 × 2 tables). Comparison of parasite prevalence between various cattle breeds was made using one-way analysis of variance (ANOVA). Comparison of the prevalence of *T. annulata* between sampling districts was calculated by chi-square test. Significance level was set at *p* ≤ 0.05.

## 3. Results

### 3.1. Prevalence of Theileria annulata in Cattle Blood Samples

*Cytochrome b* gene of *T. annulata* was targeted to report its prevalence in enrolled cattle. During the present investigation, 33% (70/210) of samples collected from District Dera Ghazi Khan and 24% (52/218) of blood samples collected from Lodhran amplified 312 base pairs specific for *cytochrome b* of *T. annulata* (Table 1).

Parasite-positive samples were further confirmed through amplification of 721 base pair fragments of *T. annulata*’s *30 kDa* gene in them (Table 1). We observed that the prevalence of *T. annulata* was significantly higher in Dera Ghazi Khan than Lodhran (Table 1) (*p* = 0.03). One-way ANOVA analysis revealed that the prevalence of *T. annulata* varied significantly (*p* = 0.04) when compared between enrolled cattle breeds, and Sahiwal cattle were most susceptible to parasite infection followed cross breed, Dajli, Jersey, Holstein Friesian and Australian, respectively (Table 2).

On the other hand, *T. annulata* prevalence varied non-significantly (*p* = 0.9) when compared between the three cattle breeds enrolled from Lodhran District (Table 2).

### 3.2. Phylogenetic Analysis of Cytochrome b and 30 kDa Merozoite Surface Antigen Gene of Theileria annulata

Four partial sequences of the *cytochrome b* gene of *T. annulata* amplified from cattle blood enrolled in the current study were confirmed by DNA sequencing and submitted to GenBank (Accession numbers: MW354912–15). Phylogenetic analysis of the amplified partial *cytochrome b* gene of *T. annulata* revealed the groups with similar sequences previously identified in cattle from India, Iran, Iraq, Tunisia, Turkey, Sudan and Egypt. The sequences reported in this study are a little further away from the *cytochrome b* gene of *T. annulata* isolated from dog (Accession number: DQ287958). Mitochondrial type homolog and *cytochrome b* gene are identified in the α-protobacteria, *Rickettsia prowazekii*. The sequence from *Rickettsia prowazekii* (Accession number: CAA74166) and those derived from protozoan parasites *Babesia bigemina* (Accession number: GQ214234) and *Babesia radhaini* (Accession number: AB624357) share less than 60% identity with our novel partial sequences, therefore they were included as outgroups for phylogenetic analysis (Figure 2).

**Figure 3:**
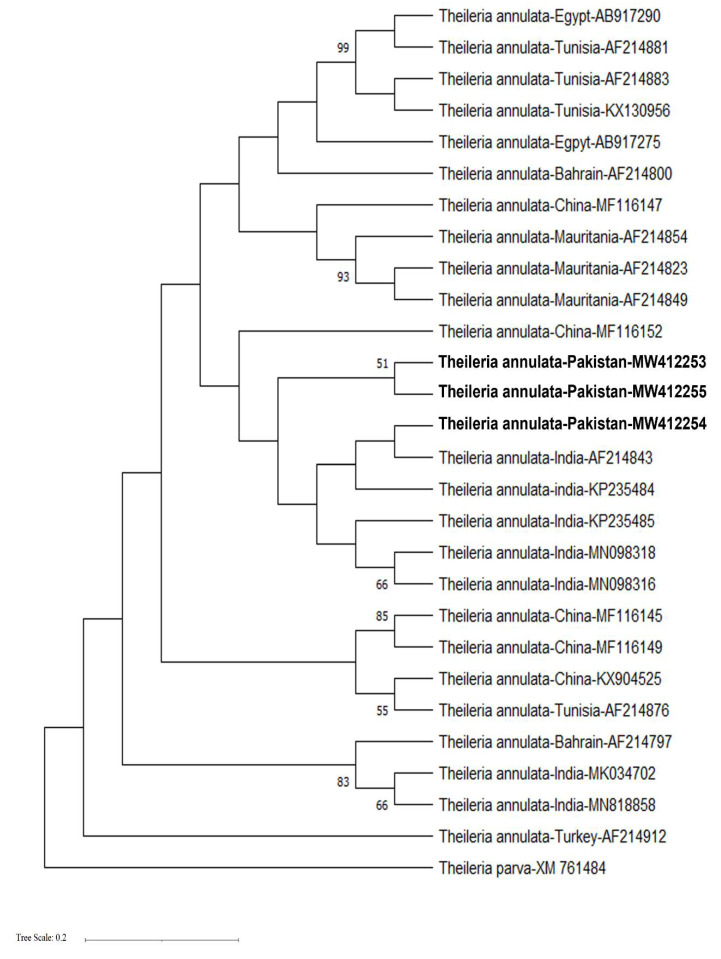
Phylogenetic tree based on partial *30 kDa* gene sequences from *T. annulata* isolates from cattle in Pakistan and worldwide, available in GenBank. The evolutionary history was inferred using the neighbour-joining and BioNJ algorithms. Three new sequences of *T. annulata* obtained in the present study are represented in bold. The scale bar represents 0.2 substitutions per nucleotide position.

Three represented amplicons of 721 bp from the *30 kDa* gene of *T. annulata* were also confirmed by DNA sequencing and submitted to the GenBank database (Accession numbers: MW412253–55). BLAST analysis revealed a nucleotide sequence match of 97–99% with the homologous sequence of *T. annulata* isolates registered in GenBank. Phylogenetic analysis revealed that our amplified sequences group together with previously identified *30 kDa* gene sequences of *T. annulata* from cattle in India and China (Figure 3).

Partial sequence from *30 kDa* gene of *T. parva* (Accession number XM_761484) and from the *Tams1* gene, an immunodominant major merozoite piroplasm surface antigen of *T. annulata* (Accession number: AF294912) were included as outgroups for phylogenetic analysis (Figure 3).

### 3.3. Analysis of Epidemiological Factors

Analysis of epidemiological factors revealed that in cattle from Lodhran, sex was the only parameter that was associated with *T. annulata* infection, and it was also found that females were more susceptible to parasites than males (*p* = 0.02) (Table 3).

For cattle enrolled from Dera Ghazi Khan, it was observed that males (*p* = 0.02), animals housed indoors (*p* = 0.04), animals with tick burden (*p* = 0.005) and farms with cattle only (*p* = 0.01) were found to be more susceptible to *T. annulata* infection (Table 3).

## 4. Discussion

Tick-borne diseases (TBDs) have been recognized as a burden to the development of the dairy industry and cause major economic losses [2,18]. During the present study, we found that 33% of cattle from Dera Ghazi Khan and 24% from Lodhran District were infected with *T. annulata* (Table 1). Among TBDs, bovine theileriosis has been frequently reported from various parts of Pakistan, and in previously documented studies, the prevalence of *T. annulata* in cattle was reported to be 33% in Peshawar and Kohat [19], 30% in Dir Upper and Chitral [20], 28% in Laki Marwat [21], 23.7% in Charsadda, Mardan and Peshawar Districts in Khyber Pakhtunkhwa [22], 21% in Layyah [23], 19% in various districts in Punjab [24] and 18.8% in three distinct zones of Khyber Pakhtunkhwa Province [25]. Similarly, the prevalence of *T. annulata* in cattle has been reported in other tropical and subtropical countries as well. The prevalence of *T. annulata* in cattle has been reported to be 23.3% in India [26], 20% in Egypt [27], 25.4% in Algeria [28], 18.2% in Northwest China [29] and 1.9% in Saudi Arabia [30]. These differences in *T. annulata* infection rates are due to variations in tick control programs, habitat suitability for ticks, farm management, husbandry practices and abiotic factors of sampling sites [20].

Genetic diversity is considered a raw material for the evolution of organisms [29]. The long-term survival of *T*. *annulata* in host animals is facilitated by the protozoan’s genetic diversity, which helps the parasite escape the host’s immune response [31]. *T. annulata* achieves this genetic diversity through chromosomal recombination in tick vectors during its sexual reproduction. In addition, genetic drift and mutation are important mechanisms to enhance their genetic diversity [3]. Thus, the acquisition of genetic diversity in parasite populations is important in establishing control measures (i.e., vaccination and drug treatments) [32]. Phylogenetic analysis is helpful in providing the basis for genetic variations and evolutionary relationships between species [30]. Over the last decade, molecular markers such as *18S rRNA, ITS1, ITS2* and *Cyt b* genes have been used to determine the phylogenetic relationship among the piroplasms population [33]. The existence of both highly conserved and variable regions of the genome, along with its universal presence, makes marker genes an important tool for determining the evolutionary relationship between organisms [34]. The data on the genetic diversity of *T. annulata* in Pakistan is very rare. In order to add to such information about this intracellular protozoan parasite, we amplified fragments from *cytochrome b* (312 base pair) and *30 kDa* genes (721 bp) of *Theileria annulata* from cattle in Pakistan and compared them with similar sequences registered in GenBank from various regions of the world. The mitochondrial *cytochrome b* gene is commonly employed for analysing phylogenetic relationships between organisms within families and genera levels [35,36,37]. The *30 kDa* antigen of *T. annulata* is a member of the major merozoite piroplasm surface antigen (mMPSA) family of polypeptides, and it is commonly targeted to report the prevalence of *T. annulata* in a variety of host animals [38]. Such studies are very significant from an evolutionary point of view as they will help in better understanding the disease patterns and the distribution of the parasite’s circulating pathogenic genotypes [14,39,40]. Phylogenetic analysis revealed sequences amplified during the present study, from both *cytochrome b* and *30 kDa* of *T. annulata,* were diverse as they were clustered with the previously reported sequences from a number of countries including India, China, Turkey, Egypt and Tunisia (Figure 2 and Figure 3). A number of biotic and abiotic factors are contributing towards these genetic variations, including the climatic and geographical conditions of sample collection sites and the prevalence of tick species involved in the transmission of parasites [41].

Analysis of epidemiological factors revealed that among all the breeds enrolled from Dera Ghazi Khan District, the Sahiwal breed was most susceptible to *T. annulata* infection (Table 2). Previously, the highest prevalence of *T. annulata* in cattle from Pakistan has been reported in exotic cattle and their crosses than the indigenous breeds [42]. Our results contradict those of Anand and Ross [43] who reported high incidences of theileriosis in crossbred cattle. Saeed et al. [42] also reported that the incidence of *T. annulata* infection was highest in crossbred cattle, followed by Cholistani and Sahiwal in the cattle blood samples collected from Dera Ghazi Khan District in Punjab. Durrani et al. [44] also reported a higher prevalence of *Theileria* spp. in crossbred cattle than in Sahiwal. On the other hand, Ndungu et al. [45] reported that different cattle breeds enrolled in their study were equally susceptible to *Theileria* infection, but there was a marked difference in their development of clinical theileriosis. differences in the parasite prevalence among cattle breeds from various studies is probably due to the different total number of samples, and due to different breed enrolment of cattle these studies.

In the present study, a higher prevalence of *T. annulata* was recorded in female cattle from Lodhran than in males from the same District (Table 3). Our results are in accordance with the findings of Inci et al. [46], Khattak et al. [22] and Saeed et al. [13], as they all reported a higher prevalence of *T. annulata* in female cattle. Saeed et al. [13] reported that comparatively weak immune response and more hormonal fluctuations in females increases the incidence of theileriosis. Kamani et al. [47] reported higher prevalence of tick-borne diseases in female cattle because they were kept longer for different purposes, such as breeding and milk production, and because they were supplied inadequate feed to satisfy their high demand.

In the present study, we observed that cattle with a tick burden were more prone to *T. annulata* infection (Table 3). A similar association between tick burden and bovine theileriosis was reported by Inci et al. [46] and Sajid et al. [48]. We observed that cattle kept indoors were more susceptible to *T. annulata* infection. This finding is in line with those of Salih et al. [49] and Farooqi et al. [24], who documented that prevalence of *T. annulata* varies with the farm management system, and that various aspects of management are potential risk factors for the spread of theileriosis.

## 5. Conclusions

In conclusion, we report that cattle from Dera Ghazi Khan District were more infected with *T. annulata* than those from Lodhran. The Sahiwal breed was found to be a most susceptible cattle breed to *T. annulata* infection. We observed that poor farm management practices are responsible for increasing TBDs in these regions. Data generated through this study will pave the way for the prophylactic detection and control of bovine theileriosis in Pakistan for the development of new vaccines and improvement in their economic output.

## Figures and Tables

**Table 1:** Comparison of *Theileria annulata* prevalence in blood samples of cattle collected from Lodhran and Dera Ghazi Khan Districts. % prevalence is shown in parenthesis. *p*-value indicates the results of chi-square test, calculated for parasite prevalence.

Sampling District	Number of Samples	*T. annulata* Positive Samples (%)	*T. annulata* Negative Samples (%)	*p*-Value
Lodhran	218	52 (24)	166 (76)	0.03 *
Dera Ghazi Khan	210	70 (33)	140 (67)	

*: Statistically significant test.

**Table 2:** Comparison of *Theileria annulata* prevalence in blood samples according to cattle breeds collected during the present study from Lodhran and Dera Ghazi Khan Districts. N represents the total number of cattle samples collected from each breed. The % prevalence of *T. annulata* is given in parenthesis. *p*-value indicates the results of one-way ANOVA estimated for the studied parameter.

	District Lodhran				District Dera Ghazi Khan	
Cattle Breed	N	*T. annulata* Positive Samples (%)	*p*-Value	N	*T. annulata* Positive Samples (%)	*p*-Value
Cholistani	99	24 (24)			-	
Sahiwal	110	26 (24)	0.9	51	23 (45)	
Crossbreed	09	02 (22)		19	8 (42)	0.04 *
Dajli	-	-		57	22 (39)	
Holstein Friesian	-	-		37	8 (22)	
Australian	-	-		35	6 (17)	
Jersey	-	-		11	3 (27)	
Total	218	52 (24)		210	70 (33)	

*p* > 0.05 = non-significant; *p* < 0.05 = statistically significant (*).

**Table 3:** Association of *Theileria annulata* infection with the studied epidemiological data of cattle enrolled from Districts Lodhran and Dera Ghazi Khan. The percent prevalence of *T. annulata* is given between parentheses. *p*-values indicate the results of Fischer exact test calculated for each parameter.

		District Lodhran (*n* = 218)			District Dera Ghazi Khan (*n* = 210)
Parameter		*T. annulata* Positive Samples (%)	*p*-Value	*T. annulata* Positive Samples	*p*-Value
Sex	Male	11 (42)	0.02 *	33 (43)	0.02 *
	Female	41 (45)		37 (28)	
Age	>5 years	28 (27)	0.3	45 (33)	0.8
	<5 years	24 (21)		25 (34%)	
Disease history	No fever	50 (24)	1	-	-
	Fever	02 (22)			
Dogs on the farm	Present	27 (21)	0.3	51 (33)	1
	Absent	25 (28)		19 (34)	
Ticks	Present	13 (27)	0.7	70 (36)	0.005 **
	Absent	39 (23)		0 (0)	
Placement of cattle	Outdoor	-	-	45 (29)	0.04 *
	Indoor			25 (45)	
Other animals	Present	-	-	54 (30)	0.01 *
	Absent			16 (53)	
Water source	Pump	-	-	27 (32)	0.7
	Pool			43(34)	

*p* > 0.05 = non-significant; *p* ≤ 0.05 = statistically significant (*, **).

## Data Availability

All the data reported in this paper has been submitted in GenBank (https://www.ncbi.nlm.nih.gov/genbank/) under accession numbers MW412253, MW412254, MW412255, MW354912, MW354913, MW354914, MW354915.

## References

[1] Hassan M.A., Liu J., Rashid M., Iqbal N., Guan G., Yin H., Luo J. (2018). Molecular survey of piroplasm species from selected areas of China and Pakistan. Para. Vect..

[2] Minjauw B., McLeod A. (2003). Tick-Borne Diseases and Poverty.

[3] Parveen A., Ashraf S., Khan A., Asif M., Iqbal F., Kumar S., Bayugar R.C., Sharma A.K., Miranda E.M., Chaubey A.K. (2021). Tick and tick-borne diseases in Pakistan. The Entomological Guide to Rhipicephalus.

[4] Jabbar A., Abbas T., Sandhu Z.U.D., Saddiqi H.A., Qamar M.F., Gasser R.B. (2015). Tick-borne diseases of bovines in Pakistan: Major scope for future research and improved control. Parasites Vect..

[5] Mans B.J.R., Pienaar A., Latif A. (2015). A review of Theileria diagnostics and epidemiology. Int. J. Parasitol..

[6] Gul N., Ayaz S., Gul I., Adnan M., Shams S., Akbar N. (2015). Tropical theileriosis and east coast fever in cattle: Present, past and future perspective. Int. J. Curr. Microbiol. Appl. Sci..

[7] Dobbelaere D., Heussler V. (1999). Transformation of leukocytes by Theileria parva and *T. annulata*. Ann. Rev. Microbiol..

[8] El Moghazy H.M., Ebied M.M., Abdelwahab M.G., El Sayed A.A. (2014). Epidemiological studies on bovine babesiosis and theileriosis in Qalubia governorate. Bent. Vet. Med. J..

[9] Watts J.G., Playford M.C., Hickey K.L. (2016). Theileria orientalis: A review. N. Z. Vet. J..

[10] Abid K., Bukhari S., Asif M., Sattar A., Arshad M., Aktas M., Ozubek S., Shaikh R.S., Iqbal F. (2021). Molecular detection and prevalence of Theileria ovis and Anaplasma marginale in sheep blood samples collected from district Layyah in Punjab Pakistan. Trop. Anim. Health Prod..

[11] Santos M., Soares R., Costa P., Amaro A., Inácio J., Gomes J. (2013). Revisiting the Tams1-encoding gene as a specific target for the molecular detection of Theileria annulata in bovine blood samples. Tick Tick-Born. Dis..

[12] Bilgic H.B., Karagenc T., Simuunza M., Shiels B., Tait A., Eren H., Weir W. (2013). Development of multiplex PCR assay for simultaneous detection of Theileria annulata, Babesia bovis and Anaplasma marginale in cattle. Exp. Parasitol..

[13] d’Oliveira C., Van der Weide M., Habela M.A., Jacquiet P., Jongejan F. (1995). Detection of Theileria annulata in blood samples of carrier cattle by PCR. J. Clin. Microbiol..

[14] Reham G.A.A., Samy S.M., Elsohabyc I., Eman A.A. (2019). Hassanena molecular and microscopical identification of bovine Theileria species isolates in Sharkia Governorate, Egypt. Egy. Vet. Med. Soc. Parasitol. J..

[15] Kumar S., Stecher G., Li M., Knyaz C., Tamura K. (2018). MEGA X: Molecular evolutionary genetics analysis across computing platforms. Mol. Biol. Evol..

[16] Hasegawa M., Kishino H., Yano T. (1985). Dating of the human-apes plitting by a molecular clock of mitochondrial DNA. J. Mol. Evol..

[17] Tamura K. (1992). Estimation of the number of nucleotide substitutions when there are strong transition-transversion and G + C-content biases. Mol. Biol. Evol..

[18] Asif M., Iqbal A., Ashraf S., Hussain M., Aktas M., Ozubeck S., Shaikh R.S., Iqbal F. (2020). First report regarding the simultaneous molecular detection of Anaplasma marginale and Theileria annulata in equine blood samples collected from Southern Punjab in Pakistan. Acta Parasitol..

[19] Khattak R.M., Rabib M., Khan Z., Ishaq M., Hameed H., Taqddus A., Faryal M., Durranis S., Gillani Q.U.A., Allahyar R. (2012). A comparison of two different techniques for the detection of blood parasite Theileria annulata in cattle from two districts in Khyber Pukhtoon Khwa province (Pakistan). Parasite J. Société Française Parasitol..

[20] Zeb J., Shams S., Din I.U., Ayaz S., Khan A., Nasreen N., Khan H., Khan M.A., Senbill H. (2020). Molecular epidemiology and associated risk factors of Anaplasma marginale and Theileria annulata in cattle from North-western Pakistan. Vet. Parasitol..

[21] Rafiullah A., Rahman K., Khan A., Ali A., Khan A., Sajid N.K. (2019). Prevalence of Theileria parva in large ruminants through conventional and molecular techniques in district Lakki Marwat and Peshawar (Pakistan). Sarhad. J. Agricul..

[22] Ullah R., Shams S., Khan M.A., Ayaz S., Akbar N., Din Q. (2021). Epidemiology and molecular characterization of Theileria annulata in cattle from central Khyber Pakhtunkhwa, Pakistan. PLoS ONE.

[23] Parveen A., Ashraf S., Aktas M., Ozubek S., Iqbal F. (2021). Molecular epidemiology of Theileria annulata infection of cattle in Layyah District, Pakistan. Exp. Appl. Acarol..

[24] Shahnawaz S., Ali M., Aslam M., Fatima R., Chaudhry Z., Hassan M., Iqbal F. (2011). A study on the prevalence of a tick-transmitted pathogen, Theileria annulata, and hematological profile of cattle from southern Punjab (Pakistan). Parasitol. Res..

[25] Farooqi S.H., Ijaz M., Saleem M.H., Rashid M.I., Ahmad S.S., Islam S., Aqib A.I., Khan A., Hussain K., Khan N.U. (2017). Prevalence and molecular diagnosis of Theileria annulata in bovine from three Districts zones of Khyber Pakhtunkhwa Province, Pakistan. Pak. J. Zool..

[26] Naik B.S., Maiti S.K., Raghuvanshi P.D.S. (2016). Prevalence of tropical theileriosis in cattle in Chhattisgarh state. J. Anim. Res..

[27] Abdel-Rady A., Kotb S., Abd Ellah M.R. (2008). Clinical, diagnostic and therapeutic studies on theileriasis (*Theileria annulata*) in cattle in upper Egypt. SCVMJ.

[28] Ayadi O., Rjeibi M.R., Elfegoun M.C.B., Gharbi M. (2016). Prevalence and risk factors of tropical theileriosis, and sequencing of Theileria annulata, the causative pathogen, in Setif region (Algeria) before and after tick season. Rev. Élevage Médecine Vétérinaire Pays Trop..

[29] Guo H., Yin C., Galon E.M., Du J., Gao Y., Moumouni P.F. (2018). Molecular survey and characterization of Theileria annulata and Ehrlichia ruminantium in cattle from Northwest China. Parasitol. Int..

[30] Alanazi A.D., Alouffi A.S., Alshahrani M.Y., Alyousif M.S., Abdullah H.H.A.M., Allam A.M., Elsawy B.S.M., Abdel-Shafy S., Alsulami M.N., Khan A. (2021). A report on tick burden and molecular detection of tick-borne pathogens in cattle blood samples collected from four regions in Saudi Arabia. Tick Tick-Borne Dis..

[31] Roy S., Bhandari V., Barman M., Kumar P., Bhanot V., Arora J.S., Singh S., Sharma P. (2021). Population Genetic Analysis of the Theileria annulata Parasites Identified Limited Diversity and Multiplicity of Infection in the Vaccine from India. Front. Microbiol..

[32] Al-Hamidhi S.H., Tageldin M., Weir W., Al Fahdi A., Johnson E.H., Bobade P. (2015). Genetic diversity and population structure of Theileria annulata in Oman. PLoS ONE.

[33] Habibi G.H. (2012). Phylogenetic analysis of Theileria annulata Infected Cell Line S15 Iran Vaccine Strain. Iran. J. Parasitol..

[34] Gupta R.S. (2016). Impact of genomics on the understanding of microbial evolution and classification: The importance of Darwin’s views on classification. FEMS Microbiol. Rev..

[35] Castresana J. (2001). *Cytochrome b* phylogeny and the taxonomy of great apes and mammals. Mol. Biol. Evol..

[36] Sharifiyazdi H., Namazi F., Oryan A., Shahriari R., Razavi M. (2012). Point mutations in the Theileria annulata *Cytochrome b* gene is associated with Buparvaquone treatment failure. Vet. Parasitol..

[37] Mhadhbi M., Chaouch M., Ajroud K., Darghouth M.A., BenAbderrazak S. (2015). Sequence polymorphism of *cytochrome b* gene in Theileria annulata Tunisian isolates and its association with buparvaquone treatment failure. PLoS ONE.

[38] Katzer F., McKellar S., Ferguson M.A., d’Oliveira C., Shiels B.R. (2002). A role for tertiary structure in the generation of antigenic diversity and molecular association of the Tams1 polypeptide in Theileria annulata. Mol. Biochem. Parasitol..

[39] Nourollahi-Fard S.R., Khalili M., Ghalekhani N. (2015). Detection of Theileria annulata in blood samples of native cattle by PCR and smear method in Southeast of Iran. J. Parasit. Dis..

[40] Kumar S., Shanker D., Paliwal S., Sudan V. (2019). Molecular characterization and sequence phylogenetic studies on Theileria annulata Mathura isolate based on TAMS and 18S gene. Ind. J. Anim. Sci..

[41] Yukari B.A., Umur S. (2002). The prevalence of tick species (Ixodoidea) in cattle, sheep and goats in the Burdur region, Turkey. Turk. J. Vet. Anim. Sci..

[42] Saeed Z., Iqbal F., Hussain M., Shaikh R.S., Farooq U., Akbar A., Gulsher M., Ayaz M.M., Mahmood S.A., Ali M. (2016). Molecular prevalence and haematology of tropical theileriosis in Cholistani cattle from nomadic herds of the Cholistan desert, Pakistan. Kafkas Univ. Vet. Faklt. Dergei.

[43] Anand D.F., Ross D.R. (2001). Epizootiological factors in the control of bovine theleriosis. Aust. Vet. J..

[44] Durrani A.Z., Mehmood N., Shakoori A.R. (2010). Comparison of three diagnostic methods for Theileria annulata in Sahiwal and Friesian cattle in Pakistan. Pak. J. Zool..

[45] Ndungu S.G., Ngumi P.N., Mbogo S.K., Dolan T.T., Mutugi J.J., Young A.S. (2005). Some preliminary observations on the susceptibility and resistance of different cattle breeds to Theileria parva infection. Onderstepoort. J. Vet. Res..

[46] Inci A.A., Ica A., Yildirim Z., Vatansever A., Çakmak H., Albasan A., Düzlü O. (2008). Epidemiology of tropical theileriosis in the Cappadocia region. Turk. J. Vet. Anim. Sci..

[47] Kamani J., Sannusi A., Eqwu O.K., Dogo G.I., Tank T.J., Kemza S., Takarki A.E., Gbise D.S. (2010). Prevalence and significance of haemoparasitic infections of cattle in North-Central, Nigeria. Vet. World.

[48] Sajid M.S., Iqbal Z., Khan M.N., Muhammad G. (2009). In vitro and in vivo efficacies of Ivermectin and Cypermethrin against the cattle tick Hyalomma anatolicum anatolicum (Acari: Ixodidae). Parasitol. Res..

[49] Salih D.A., Hussein A.E., Kyule M.N., Zessin K.H., Ahmed J.S., Seitzer U. (2007). Determination of potential risk factors associated with Theileria annulata and Theileria parva infections of cattle in the Sudan. Parasitol. Res..

